# Maternal microchimeric cell trafficking and its biological consequences depend on the onset of inflammation at the feto-maternal interface

**DOI:** 10.1007/s00281-025-01037-w

**Published:** 2025-01-17

**Authors:** Emiel Slaats, Bernadette Bramreiter, Kristine J. Chua, Rachel C. Quilang, Katja Sallinger, Michael Eikmans, Thomas Kroneis

**Affiliations:** 1 Gottfried Schatz Research Center, Division of Cell Biology, Histology and Embryology, Medical University of Graz, Graz, Austria; 2 Department of Anthropology, University of California Santa Barbara, Santa Barbara, CA USA; 3 Department of Anthropology, University of Notre Dame, Notre Dame, CA USA; 4 Department of Immunology, Leiden University Medical Center, Leiden, The Netherlands

**Keywords:** Microchimerism, Inflammation, Labor, Pregnancy, Spatio-temporal corridor

## Abstract

Microchimerism is defined as the presence of a small population of genetically distinct cells within a host that is derived from another individual. Throughout pregnancy, maternal and fetal cells are known to traffic across the feto-maternal interface and result in maternal and fetal microchimerism, respectively. However, the routes of cell transfer, the molecular signaling as well as the timing in which trafficking takes place are still not completely understood. Recently, the presence of inflammation at the feto-maternal interface has been linked with maternal microchimeric cells modulating organ development in the fetus. Here, we review the current literature and suggest that inflammatory processes at the feto-maternal interface tissues are a physiological prerequisite for the establishment of microchimerism. We further propose a spatio-temporal corridor of microchimeric cell migration to potentially explain some biological effects of microchimerism. Additionally, we elaborate on the possible consequences of a shift in this spatio-temporal corridor, potentially responsible for the development of pathologies in the neonate.

## Introduction

In the past few decades, several groups identified nonself cells to be present in host tissues and capable of persisting for a lifetime [1–3]. This phenomenon is referred to as microchimerism (MC), defined as the presence and persistence of a small number of cells in a host that originate from a genetically different individual. The etiology of MC can be artificially introduced by transplantations [4], or naturally occurring due to the bidirectional trafficking of cells during pregnancy [2]. Based on the latter, two main sources of MC are described, maternal cells that home to fetal tissues (giving rise to maternal MC; mMC) or fetal cells that transfer to maternal tissues (fetal MC; fMC) [1]. However, the concept of MC is not restricted to the mother-child dyad as sibling, grandmaternal, and MC of unknown origin have also been reported [5–7]. The biology of MC has been linked with several beneficial and harmful effects including tissue repair, wound healing, immune system development, cancer, and autoimmune diseases (reviewed in [8, 9]). Recent research has begun to provide insights into how MC cells travel throughout the body as seen through transplacental gradients [10] and homing pathways within the host [11, 12]. In the case of mMC, it has been shown that maternal cells can reach the fetus as early as the second trimester of pregnancy in mice [13] and humans [14], with mMC levels peaking around birth [15]. In addition, maternal cells have been shown to continue trafficking to the newborn during nursing [16]. However, the specific routes and processes involved in cell trafficking still remain unclear and long for additional evidence. Recently, Ward and colleagues showed how preterm placental inflammation impacts the placental barrier function and leads to a maldevelopment of the murine embryonic heart by a maternal monocyte mediated mechanism [17]. The process by which maternal cells cross the placental barrier during challenged and unchallenged pregnancies is still unknown. Furthermore, the association between impaired brain development and maternal inflammation in humans and mice have also been observed [18]. Inflammation-associated preterm labor in mice correlates with increased trafficking of maternal cells [19]. But, unlike the case of maldevelopment of the heart, migrating maternal CD8^+^ T cells appear to have caused the perinatal brain injury as their depletion rescued the phenotype [20].

In humans, inflammation at the feto-maternal interface has recently been described in four out of five healthy term deliveries with only 1% of the cases showing severe placental lesions [21]. Due to the lack of obstetric complications and the low number of severe lesions, mild inflammatory lesions may go unnoticed in the clinical daily routine. Considering the large number of tested pregnancies, these inflammatory processes seem likely to represent a widespread phenomenon during pregnancy. This underlines the general importance of inflammatory processes in the context of parturition and thus, human reproduction [22].

**Fig. 1:**
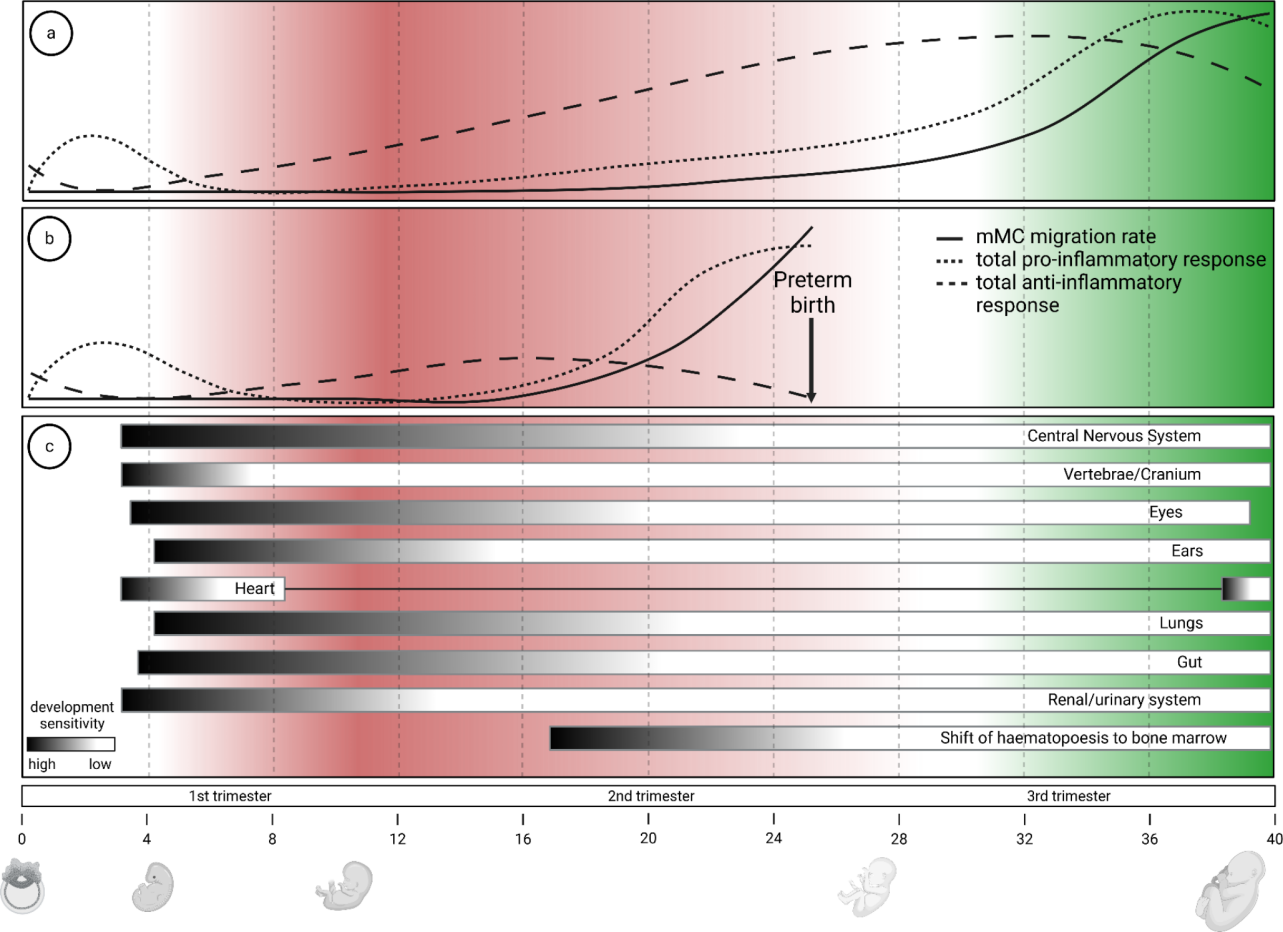
The spatio-temporal corridor of maternal microchimerism **a** Throughout gestation, a physiological balance of pro-inflammatory (dotted line) and anti-inflammatory (dashed line) responses at the feto-maternal interface secures tolerance of the fetus. Maternal cells traffic to the fetus throughout gestation. However, at the end of pregnancy, inflammatory processes increase, causing increased attraction of cells to the feto-maternal interface and the initiation of labor and parturition. This inflammatory process causes a boost in maternal cell trafficking at term, i.e., within the physiological (green) corridor for mMC. **b** The shift in balance to a pro-inflammatory environment can be moved towards an earlier gestational week, e.g., due to an infection. Increased inflammation at the feto-maternal interface results in elevated maternal cell trafficking now outside the physiological corridor (red). **c** Temporal changes in trafficking grants MC cells access to still developing and maturating embryonic and fetal tissues, which may impact these processes. The mMC influx can take place during a critical (black) or less sensitive phase (white) of development or maturation, thereby dictating the risk of developing a pathology in specific organs

While an early transfer of maternal cells has been proposed to mainly modulate organ development and maturation, a transfer of maternal cells later in pregnancy might be associated with additional beneficial immunological effects [23]. From an evolutionary perspective, the transfer of maternal cells to the fetus just before parturition seems to be the optimal window for the mother to expand her immunological protection of the offspring beyond her womb. In doing so, fitness benefits may arise where mothers transmit immunological support to ensure the survival of their offspring well into the next generation in vertebrates, including humans, and invertebrates [24, 25]. As such, evolution is thought to favor the perinatal trafficking of cells, likely maternal microchimeric cells. They could offer an initial short-term immunoprotection for the offspring post-delivery [26], which can be even further extended via breastfeeding [16, 27, 28]. Since placental inflammation, a widespread physiological process involved in labor and parturition, has been linked to mMC, we may have reason to suspect maternal microchimeric cell trafficking to be similar in frequency and time course with the latter, impacting the biological consequences of MC. Based on the reviewed literature, we propose a spatio-temporal model of maternal cell migration as an important physiological process in human reproduction. This model proposes that a population of maternal cells colonizes feto-maternal interface tissues just prior to and during parturition, utilizing this inflammatory process in order to traffic to the fetus. In healthy-term pregnancies (Fig. 1a), this process parallels the shift to a pro-inflammatory state leading up to labor, provides immunological benefits and generally remains undetected. However, if this process is shifted in time (Fig. 1b), due to an infection that induces a pro-inflammatory environment for example, those maternal cells gain early access to fetal tissues that are still undergoing development and maturation, which causes a time- and site-dependent impact (Fig. 1c).

## MC cells are detected along the border tissues of the feto-maternal interface in healthy pregnancies

**Fig. 2:**
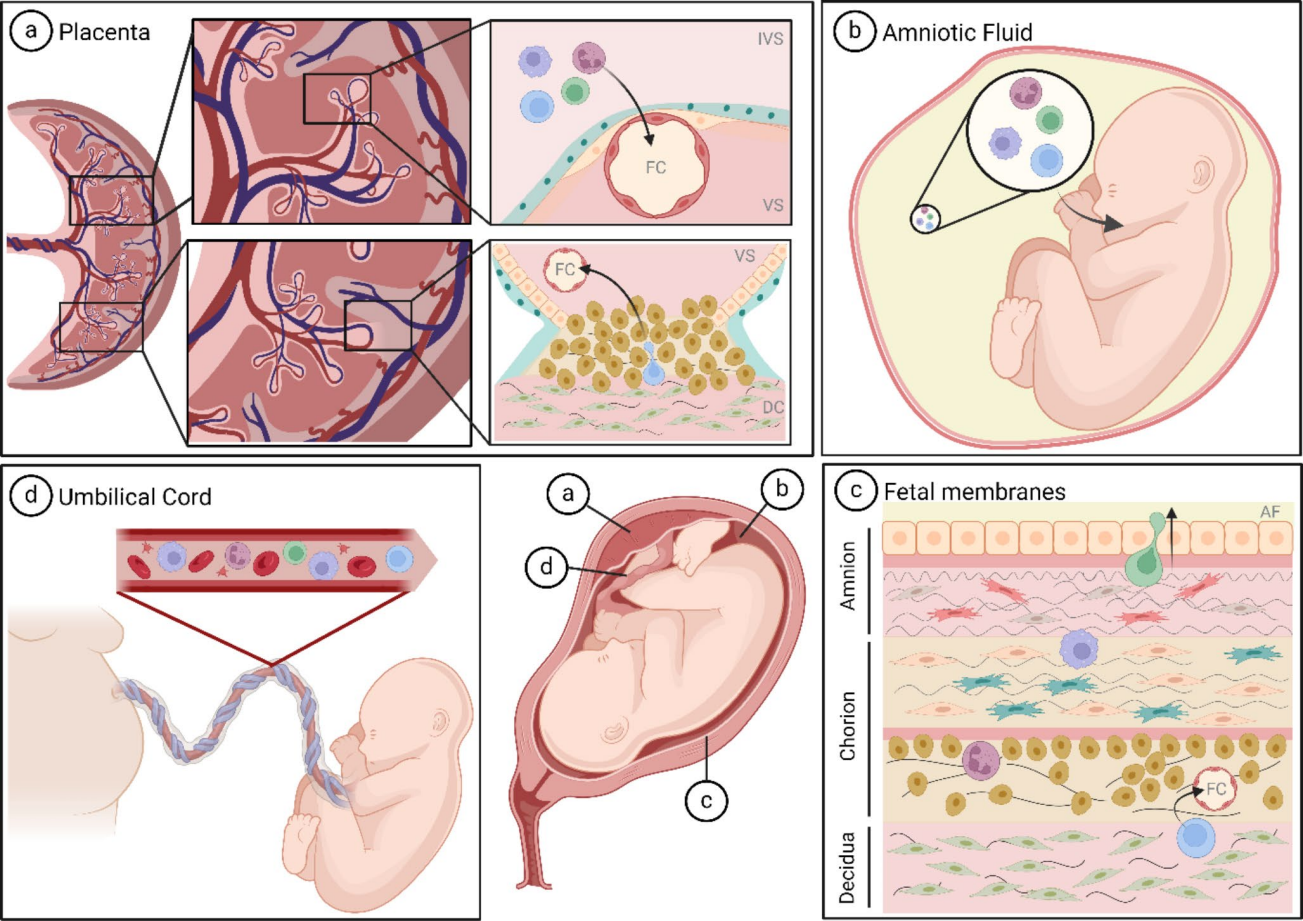
Proposed routes for maternal cells to traffic from mother to fetus during pregnancy. **a** Upper panel: Maternal immune cells migrate from the intervillous space (IVS) across the syncytiotrophoblast and cytotrophoblast cells of a terminal villus and intravasate a fetal capillary (FC) situated in the villous stroma (VS) to enter the fetal circulation. Lower panel: Maternal cells located in the decidua (DC) basalis bypass the cytotrophoblast layer of an anchoring villus and migrate towards a fetal capillary (FC) to get access to the fetal circulation. **b** Free floating maternal cells in the AF are swallowed and inhaled by the fetus. Upon intake, the maternal cells subsequently need to transmigrate epithelial layers of the relevant tissues to gain access to the fetal circulation (not shown). **c** Maternal immune cells traffic from the decidua parietalis to the chorionic membrane and transmigrate the endothelial layer of a fetal vessel gaining access to the fetal blood. Some other maternal cells cross the chorionic and amniotic membranes to enter the amniotic fluid (AF). **d** Maternal cells that managed to intravasate extra-embryonic capillaries in the placental villi (Fig. 2a) and chorionic membrane (Fig. 2b) get access to the fetal circulation via the umbilical cord

The underlying processes of maternal cells trafficking to fetal tissues are still largely unknown. However, several routes have been described and for some theoretical routes, we currently lack experimental evidence. In this section, we will provide an overview of the routes (summarized in Fig. 2) maternal cells have been identified under uncomplicated term pregnancy conditions. Recently, maternal cells have been reported to be capable of trafficking a trans-placental route. They used isolated cells from maternal circulation to perfuse the placenta ex vivo and identified maternal immune cells attach to the syncytiotrophoblast layer, cross the placental barrier, and migrate into the fetal villous mesenchymal stroma [29, 30]. Once close to a fetal vessel, the microchimeric cells would need to intravasate to get access to the fetal circulation via the umbilical vein (Fig. 2a and d). A theoretical modification of this trans-placental route involves the recruitment of maternal cells to the decidua basalis where they migrate into the villous stroma and capillaries via the attachment points of the anchoring villi (Fig. 2a). Alternative to the trans-villous route, maternal cells might also travel to the fetus via the fetal membranes. Following this concept, maternal cells migrate from the decidua parietalis into the chorionic membrane. Once there, they get access to the fetal circulation similar to the process in the placental villi (Fig. 2c and d). A modification of this route comprises maternal cells trafficking across the amniotic membrane and entering the amniotic fluid (AF; Fig. 2b and c). Colonizing the AF, cells could enter the fetal body via the gastro-intestinal tract upon swallowing the AF (Fig. 2b), which starts at around gestational week 13 [31]. Theoretically, cells could also get access to the fetal airways when AF is inhaled perinatally. Cells migrating via the AF-mediated route subsequently need to undergo transepithelial migration, as has been described for maternal cells transferred during nursing in mice [16, 32]. Importantly, evidence for the presence of maternal cells has been reported in several tissues including deciduae, chorioamniotic membranes, placental villi, and cord blood at the end of the third trimester and at birth. These potential migratory routes are in no way mutually exclusive and may differ between cell types, timepoints, and overall pregnancy status. Several maternal cell types are present in the third trimester placental villi of uncomplicated pregnancies. Single-cell transcriptomics have revealed that maternal naïve CD4^+^ and CD8^+^ T cells, activated CD8^+^ T cells, monocytes, macrophages, B cells, and plasma cells are located in the placental villi [33, 34]. Furthermore, fluorescent in situ hybridization analyses using X and Y chromosome probes (XY-FISH) and short-tandem repeat DNA profiling have shown that rare but viable, maternal mesenchymal stem cells (MSCs) are located in the villous stroma of mature placentas [35]. Interestingly, injected human MSCs have been shown to be capable of crossing the placenta and invading fetal tissues in rats [10], suggesting MSCs to be an interesting candidate also in the human environment. Further support for the active migration of maternal cells is provided by Morales-Prieto and colleagues. They detected maternal T and natural killer (NK) cells in the villous stroma as well as in fetal capillaries of perfused term placentas [30]. In chorioamniotic membranes at birth, Pique-Regi and colleagues determined maternal T cells, NK cells, macrophages, and monocytes to be the major cell populations [33]. Furthermore, their data also seems to suggest a low presence of maternally derived B cells [33]. Near parturition, maternal memory-like T cells get actively recruited to the chorion-decidua [36], under influence of a physiological inflammatory environment [22]. Interestingly, T cells, potentially of maternal origin, have been reported to infiltrate the fetal membranes near the rupture zone at birth [37]. In cord blood collected at birth, mMC is most prevalent in memory T and naïve T cell compartments. Furthermore, maternal cells have been shown to contribute to NK cells, B cells, and monocyte/granulocyte populations [7, 38–40]. In addition, the presence of maternal hematopoietic stem and progenitor cells has been reported at variable concentrations. The relevance of maternal cells in cord blood to long term MC has been proven by their longitudinal detection in patients transplanted with cord blood [39]. The presence of maternal cells in the cord blood supports the existence of trans-endothelial processes representing a subsequent step towards mMC. Although the literature is scarce, maternal stem and immune cells have been detected along the different proposed routes in uncomplicated term pregnancies. Identical populations have been found and identified to be of maternal origin in placental villi, chorioamniotic membranes, and cord blood. Based on the data we may assume oncoming labor to be the onset for a time farme allowing (increased) maternal cells to traffic and home as MC cells to fetal tissues.

## Inflammation in feto-maternal interface tissue is a physiological process in parturition and facilitates MC cell trafficking

Several mechanisms have been reported to be involved in the triggering of labor and parturition. Among the viviparous species, at least three main processes are widely shared at the end of pregnancy: contractions of the myometrium, softening of the cervical extracellular matrix, and inflammation of the feto-maternal interface tissues [22]. Of these processes, inflammation provides the most intriguing concept with respect to mechanisms of cellular transfer giving rise to mMC. Representing the maternal part, the decidua can contribute to adjusting the type and function of residing immune cells and modulating inflammatory signals that are linked with initiating parturition [22]. During early pregnancy, mesenchymal cells in the decidua are able to attract and differentiate a range of immune cells of the myeloid and lymphoid lineage by using interleukins, chemokines, and TGF-ß [41–43]. The attraction also includes cells from the periphery, such as activated T cells and dendritic cells [44–47]. Later in pregnancy, the decidual cells express TNF-α and IL-1 and shift to an inflammatory phenotype, thereby contributing to uterine contractions [48]. Furthermore, they are capable of regulating tryptophan metabolism, which is associated with feto-maternal tolerance [49, 50]. Especially IDO1, the key enzyme in tryptophan metabolism, was shown to be crucial for maternal immune tolerance [51]. Interestingly, IDO1 expression was also found at the fetal side in endothelial cells underneath the cytotrophoblast cells, suggesting an IDO1 gradient towards the feto-maternal interface [52]. Thus, inflammation-induced trafficking maternal microchimeric cells might be moving in a tolerogenic environment until entering the fetal circulation to prevent complications. The inflammatory process is not restricted to the decidua, but also occurs within the AF upon sterile inflammation during spontaneous preterm labor [53]. This links fetal and maternal inflammation with parturition suggesting that feto-maternal inflammation might be a physiological process during labor and parturition. However, infections and environmental signals potentially disturb the highly regulated interplay between inflammation and parturition and can cause a shift toward preterm deliveries [54–57]. In addition, parturition is supported from the fetal side as inflammation of the chorion has been described and linked with preterm birth in subsequent pregnancies [58]. Recently, Romero and colleagues screened 944 pregnancies with normal outcomes and identified placental lesions in 78% of them. Women in labor showed acute inflammation most frequently [21]. Despite the high percentage of inflammatory events, very few (1%) of the lesions turned out to be severe. As it did not affect the clinical course, these local lesions seem to contribute to a physiological inflammatory process. A second important process common among viviparous species is the softening of the cervical tissues. This softening goes along with the activity of matrix-metalloproteinases (MMPs) that are known to break down molecules of the extracellular matrix allowing for the migration and invasion of proteins [59]. They can be activated in decidual cells upon mechanical stress [60] and are especially involved in the rupture of membranes under several conditions including bacterial infection and sterile inflammation [61]. MMPs are present beyond the decidua in AF and fetal membranes throughout pregnancy, and their association with membrane destruction and rupture is linked to term as well as preterm delivery [62–67]. Interestingly, MMPs have been reported to act on a cell’s environment via extracellular vesicles helping cells maintain their invasiveness [68, 69]. Recently, Okusha and colleagues reported increased invasion of recipient cells in an allograft mouse model after an uptake of MMP3-positive extracellular vesicles in host tissue [70]. MMP’s orchestrated activity around parturition, pro-invasive effect of matrix degeneration, and potential to modify the host tissue via extracellular vesicles suggest that they play a role in the trafficking of MC cells. While the shift to a pro-inflammatory environment leading up to labor ‘opens the door’ for potential transplacental migration of cells, this period is of particular interest from an evolutionary perspective because of the drastic shift in environment between the uterus and the outside world. The fetus must prepare itself before birth for microbial colonization, pathogen exposure, and uncontrolled commensal species that are not present *in utero* [24]. Because immunocompetent maternal immune cells retain immunological memory of previously encountered pathogens in the maternal environment [24], maternally derived cells that are transferred to the fetus can aid the fetus’ immune defense. For instance, circulating maternal microchimeric cells in children whose mothers had been infected with placental malaria, have immune systems primed to respond to *P. falciparum*; more frequent infections but less severe clinical manifestations [71]. Similarly, recent work detecting T cell clones against common pathogens in villitis of unknown etiology (VUE) lesions [72] along with the detection of maternal pathogen-specific T cells in a preconception infection mouse model [27] and human severe combined immunodeficiency (SCID) patients [26] further supports this notion. With the omnipresence of inflammation at labor and parturition, cellular invasion of the interface tissues may be seen as a prerequisite to their trafficking beyond the placenta. Thus, the inflammatory process in pregnancy might be one of the mechanisms involved in establishing MC. Furthermore, we argue that this perinatal trafficking of maternal microchimeric immune cells to the fetus prior to labor is essential not only for *in utero* protection but also for building an effective immune response in the offspring. Evolution may have led to favoring the transfer of these immunocompetent cells and thereby offer a more robust immune system for the fetus [25]. However, the timing of when these cells migrate is important, as we argue that acquiring these cells is only adaptive if it occurs prior to labor. The unintended activation of maternal immune cells earlier in gestation could disrupt developmental processes, increasing the risk of pathology [73]. Damages incurred by this dysregulation can harm both the mother and her offspring, ultimately decreasing fitness [23]. Taken together, we propose labor to be a widespread physiological inflammatory process recruiting maternal (immune) cells to the interface tissues enabling them to transmigrate into the fetus (spatio-temporal corridor; Fig. 1). However, this raises the question whether inflammation at the feto-maternal interface occurring earlier during pregnancy opens an additional window for establishing MC.

## Inflammatory lesions enable MC cell trafficking independently from parturition

As indicated above, a body of evidence supports the presence of maternal cells in the relevant feto-maternal interface tissues at the end of pregnancy. Furthermore, the inflammatory environment leading up to labor might facilitate physiological cell trafficking. In mice, it has been shown that inflammation at the feto-maternal interface causes increasing numbers of maternal cells to traffic to the offspring. In particular, the migration of maternal T cells and monocytes/granulocytes was increased [19, 74]. If this holds true for humans, it should be expected that this is reflected in an increase of maternal cells at sites of (pathological) placental inflammation. One of the most common sterile inflammatory lesions is VUE. These lesions are characterized by a lymphocytic infiltrate in the placental villi predominantly consisting of maternal CD4^+^ and CD8^+^ T cell types. In addition, there is a minor contribution of maternal cells among the CD68^+^ macrophages in the mesenchymal stroma of the villi. B cells, potentially of maternal origin, can be present at very low levels [72, 75–80]. In uncomplicated term placentas, VUE lesions are typically detected with a frequency of up to 15% [81]. However, in complicated pregnancies, the frequency can be significantly increased. High-grade VUE is associated with fetal growth restriction, intrauterine fetal death, and cerebral palsy [82–84]. Chronic basal villitis (CBV), a subtype of villitis involving the villi bordering the basal plate, is present in about 4% of placentas [85]. CBV is often co-occurring with chronic deciduitis, which is reflected by the co-invasion of decidua-derived plasma cells and CD4^+^ and CD8^+^ T cells, typically present in inflammatory lesions in case of villitis [86]. Those plasma cells are presumed to be of maternal origin. However, experimental verification is still needed. Chronic chorioamnionitis (CCA) represents another common inflammatory lesion. It is often co-occurring with VUE [87] and is characterized by maternal CD8^+^ T cells infiltrating the chorionic and amniotic membranes [88]. Furthermore, CCA is linked to preterm labor and premature rupture of membranes [87]. Typically, CCA is detected in 10% of all term placentas [89]. Unlike in VUE, acute/infectious villitis (AIV) is diagnosed upon proven pathogen involvement. This translates to differences in immune infiltrates as well. While maternal CD4^+^ and CD8^+^ T cells might be present, AIV lesions are characterized by a massive presence of maternal neutrophils and/or plasma cells [90], which are absent in VUE [81]. Acute chorioamnionitis, like AIV, is characterized by a massive infiltration of maternal neutrophils, but only in the chorionic and amniotic membranes. An important characteristic of acute chorioamnionitis is the involvement of the AF, as the inflammation spreads into the amniotic cavity and triggers an immune response in the AF. The immunophenotype of inflamed AF includes innate lymphoid cells (ILCs), NK cells, B cells, T cells, monocytes, and neutrophils [91]. Furthermore, elevated IL-6 levels and microbial colonization can be detected. Neutrophils and macrophages can be of maternal or fetal origin, or both. Interestingly, a predominance of fetal neutrophils and macrophages is associated with early and late preterm birth, while a predominance of immune cells of maternal origin rather results in late preterm or term birth [92, 93]. The infectious inflammatory lesions in the placenta have been associated with diverse adverse neonatal outcomes. The clinical associations include preterm birth, pulmonary defects, heart defects, deafness, blindness, and neurological defects [90, 94–96]. Considering that some of the populations of invading maternal cells described above are commonly detected in cord blood samples [7, 39, 40], it is likely that the invading cells from feto-maternal lesions travel further into the offspring. This is especially relevant considering that Labarrere and colleagues identified maternal cells in both the villous stroma and villous capillaries of VUE and in massive chronic intervillositis [76]. Furthermore, mMC levels are increased in cord blood of preterm births [97], a pregnancy complication strongly connected to inflammation at the feto-maternal interface. Moreover, mMC has also been reported to be increased in the cord blood of infants with inflammatory placental malaria [71]. Since intervillositis, a mononuclear cell infiltrate of the intervillous space, is very common in placental malaria [98], this fits well with the report of Labarrere mentioned before. Taken together, placental inflammation seem to be quite common in human pregnancies suggesting another link to the establishment of mMC. This link, however, would include non-physiological inflammation which has the potential to alter the timeframe of maternal immune cell trafficking. Moreover, the consequences of such alterations in the corridor are still unknown.

## Altered MC migration modulates fetal development and maturation

Over the course of the previous sections, we introduced the idea of a physiological inflammatory state leading up to labor. As such, it might frame a spatio-temporal corridor of major MC cell transfer (Fig. 1a). If, however, excessive inflammation occurs due to infection or other environmental stressors (Fig. 1b), migration of immune competent cells shifts outside the defined spatio-temporal corridor with various potential effects. In the case of an infection, maternal pathogen specific cells might be transferred, promoting immune development and providing immune protection for the offspring, thereby increasing their survival. In mice, the transfer of pathogen specific T cells that increase the offspring’s fitness has been described [27]. This is further illustrated by the case report of the expansion of Epstein-Barr virus specific maternal microchimeric cells in a human SCID patient [99]. However, since maternal cells have been implicated in fetal monocyte development [100] as well as lowering T and NK cell activation [101] in mice outside of infection, potentially disadvantageous alterations in postnatal immune function might also be expected. This is especially the case when the inflammatory environment is not pathogen mediated. As it could be speculated to increase the susceptibility to allergies and autoimmune reactions. Furthermore, the specific magnitude, type, and location of the inflammatory response might also result in the migration of cells that under the condition of a physiological pregnancy, would not migrate. Moreover, trafficking maternal microchimeric cells could have altered activation states and/or specificity. As a result of all these deviations, the fetus may receive maternal cells that it is unable to properly process. Consequently, the maternal cells might home to sites where they are not intended to go. Depending on the timepoint, the likely consequence is that processes like organ development or maturation are disturbed, with organ-specific pathology in the offspring (Fig. 1c). However, due to the high variety in immunologic insults, genetic background and time point of excessive inflammation, as well as medical treatment in humans, it will be very challenging to explore these effects. To further complicate this matter, it should be acknowledged that inflammation can affect the developing fetus by independent processes that parallel increased maternal microchimeric cell transfer. Examples include the transplacental transfer of inflammatory cytokines [102] or antibodies [103]. However, in the existing literature, there is support for the suggestion that a shift in the spatio-temporal corridor can lead to pathology. For example, inflammation induced migration of maternal monocytes disturbs heart development in mice [17]. Interestingly, in humans, increased mMC was detected in the hearts of some fetuses with neonatal lupus congenital heart block [104], which is a condition associated with systemic lupus erythematosus, an autoimmune disease, in the mother. Additionally, increased inflammatory markers and mMC in cord blood seem to correlate with increased severity of congenital diaphragmatic hernia in humans [105]. Furthermore, since altered mMC has been linked to impaired brain development and behavior in mice [106], our conceptual framework might partly explain the strong association between maternal inflammation and impaired cognitive development that has been observed in humans and mice [18, 107]. In support, mMC has been reported to be increased in cord blood of infants with inflammatory placental malaria [71]. Importantly, malaria infection during pregnancy, like other infections, is implicated in impaired neurodevelopment [108]. On a final note, based on the proposed spatio-temporal concept (Fig. 1), there are some other neonatal pathologies that we could speculate might have mMC involvement. One example might be lung defects since induced chorion amniotic inflammation (infection and other) has been linked to a disruption of lung development [109]. Furthermore, it might be speculated that inappropriate maternal cell migration, in part, also contributes to neonatal sight and hearing impairment as seen as a consequence of congenital Toxoplasma infection [96]. At least some Toxoplasma infections are accompanied by AIV lesions with maternal cell infiltrates [110]. Lastly, lipopolysaccharide induced chorioamnionitis in a sheep model has been shown to promote inflammation and damage to the neonatal kidney [111]. Based on our spatio-temporal concept, maternal recruitment might be expected. However, a significant amount of experimental work will need to be carried out to test these hypotheses.

## Histocompatibility, a potential modulator influencing the spatio-temporal corridor concept

Coming back to the previously mentioned point of genetic background influencing the spatio-temporal corridor, it is important to consider if compatibility between the mother child dyad could modulate physiological and pathological effects of mMC. As there are many factors that can influence mMC throughout infancy, this section will highlight HLA compatibility or incompatibility in the context of autologous pregnancy and oocyte donation (OD) pregnancy. Studies have shown that maternal compatibility at class II DQB1 and possibly DRB1 HLA loci favors mMC, which is not the case for class I. Interestingly, there seems to be a relation with the mother carrying the HLA-DQB1*0301 allele as well [112]. Although this topic is highly complex, future insights might be gained from OD donation pregnancies. OD is an assisted reproductive technology procedure that enables women with several causes of infertility to conceive [113]. However, due to the higher degree of antigenic dissimilarity between mother and fetus during OD pregnancies compared with non-OD pregnancies, there is an increased risk for pregnancy complications [114, 115]. In OD pregnancies, decidual maternal cells are in contact with highly HLA-mismatched fetal cells and it is thought that these decidual maternal cells play a crucial role in the maintenance of healthy uncomplicated OD pregnancies [116]. Interestingly, a recent study observed that uncomplicated OD pregnancies display the highest incidence of placental inflammatory lesions where the offspring was fully allogeneic [117]. However, it was found that the maternal immune infiltrate had more of an immunosuppressive role, as these cells were expressing IL-10, which could protect the allogeneic fetus in case of highly mismatching HLA types. This was also supported by Schronkeren and colleagues who found a specific maternal macrophage infiltrate in the chorionic plate to be associated with improved pregnancy outcomes in OD pregnancies [118]. It is unclear if the degree of immunogenetic dissimilarity between mother and child in OD pregnancy has an effect on the frequency of mMC [119] as in both studies, mMC was not investigated. Therefore, one could only speculate if the maternal immune infiltrate to the placenta also trafficked to the fetus to continue exerting an immunoprotective role. Even though human studies on the effect of mMC in OD pregnancies are lacking, a major study by Kinder and colleagues, showed how antigen compatibility and resulting mMC improved trans-generational fitness in a murine model [120]. They found that persistent maternal microchimeric cells in female offspring were required in order to maintain Treg cell non-inherited maternal antigen (NIMA)-specific tolerance. Furthermore, female offspring with NIMA-specific Tregs showed improved reproductive outcomes when mated with males expressing overlapping MHC haplotypes, thus promoting reproductive fitness in the next generation [120]. This further highlights the need to experimentally examine histocompatibility in the context of the proposed spatio-temporal corridor.

## Conclusion

In conclusion, we propose that maternal cell trafficking to the offspring is, at least in part, a direct consequence of inflammation along the feto-maternal interface. In uncomplicated pregnancies, the physiological inflammation that induces labor facilitates the timed transfer of a population of maternal immune cells that will assist in developing the offspring’s immune system after birth. If this population of maternal cells travels outside of this defined time frame as a consequence of inflammation due to infection or other environmental insults, there is an increased risk of MC mediated pathology in the neonate. Although we focused on mMC in this review, it would be interesting to explore if and how the proposed concepts might translate to fetal derived MC in the mother.

## Data Availability

This manuscript has no associated data available.
